# *Bacillales*: From Taxonomy to Biotechnological and Industrial Perspectives

**DOI:** 10.3390/microorganisms10122355

**Published:** 2022-11-28

**Authors:** Sharareh Harirchi, Taner Sar, Mohaddaseh Ramezani, Habibu Aliyu, Zahra Etemadifar, Seyed Ali Nojoumi, Fatemeh Yazdian, Mukesh Kumar Awasthi, Mohammad J. Taherzadeh

**Affiliations:** 1 Swedish Centre for Resource Recovery, University of Borås, 50190 Borås, Sweden; 2 Microorganisms Bank, Iranian Biological Resource Centre (IBRC), Academic Center for Education, Culture and Research (ACECR), Tehran, Iran; 3 Institute of Process Engineering in Life Science II: Technical Biology, Karlsruhe Institute of Technology, 76131 Karlsruhe, Germany; 4 Department of Cell and Molecular Biology & Microbiology, Faculty of Biological Science and Technology, University of Isfahan, Isfahan 8174673441, Iran; 5 Microbiology Research Center, Pasteur Institute of Iran, Tehran 1316943551, Iran; 6 Department of Mycobacteriology and Pulmonary Research, Pasteur Institute of Iran, Tehran 1316943551, Iran; 7 Department of Life Science Engineering, Faculty of New Sciences and Technologies, University of Tehran, Tehran 1439957131, Iran; 8 College of Natural Resources and Environment, Northwest A&F University, Taicheng Road 3#, Yangling, Xianyang 712100, China

**Keywords:** *Bacillales*, *Caryophanales*, taxonomy, biotechnology, engineered strains, extremophiles

## Abstract

For a long time, the genus *Bacillus* has been known and considered among the most applicable genera in several fields. Recent taxonomical developments resulted in the identification of more species in *Bacillus*-related genera, particularly in the order *Bacillales* (earlier heterotypic synonym: *Caryophanales*), with potential application for biotechnological and industrial purposes such as biofuels, bioactive agents, biopolymers, and enzymes. Therefore, a thorough understanding of the taxonomy, growth requirements and physiology, genomics, and metabolic pathways in the highly diverse bacterial order, *Bacillales*, will facilitate a more robust designing and sustainable production of strain lines relevant to a circular economy. This paper is focused principally on less-known genera and their potential in the order *Bacillales* for promising applications in the industry and addresses the taxonomical complexities of this order. Moreover, it emphasizes the biotechnological usage of some engineered strains of the order *Bacillales*. The elucidation of novel taxa, their metabolic pathways, and growth conditions would make it possible to drive industrial processes toward an upgraded functionality based on the microbial nature.

## 1. Introduction

*Bacillales* (later heterotypic synonyms of *Caryophanales*) is the most productive order of the phylum *Firmicutes*. The enormous diversity of the order, which includes numerous genera and species, resulted in scanty detailed studies of the diverse taxa. Based on 16S rRNA gene sequencing analysis, phylogenomics, and other approaches, *Bacillales* is delineated to include ten validly published families [1]. Of these, the family *Bacillaceae* comprises certain strains capable of surviving under various conditions, which, from an anthropogenic perspective, are considered extreme, including high or low temperatures and pH ranges and high salt concentrations. In addition to these extremes individually, some members of *Bacillaceae* can survive under multiple combinations of the above extremes and, hence, are considered polyextremophiles [2]. The ability to withstand harsh conditions placed members of the order *Bacillales* at the center of interest for various industrial applications, and [3] up to now, additional biotechnologically relevant insights are being gained, even among the well-known species and strains.

Recent advances in novel molecular techniques, omics approaches, and modern data processing and sharing capabilities enable us to expand our knowledge toward other less-known genera, species, and strains. Despite these, most of the contemporary literature on *Bacillaceae* focuses on a few select strains from the genus *Bacillus*, pathogenic genera, and some *Bacillus* related-genera. These reviews are enlightening, but none of them comprehensively discuss various genera belonging to the order *Bacillales* that, in turn, can be considered novel candidates for biotechnological and industrial applications.

**Figure 1:**
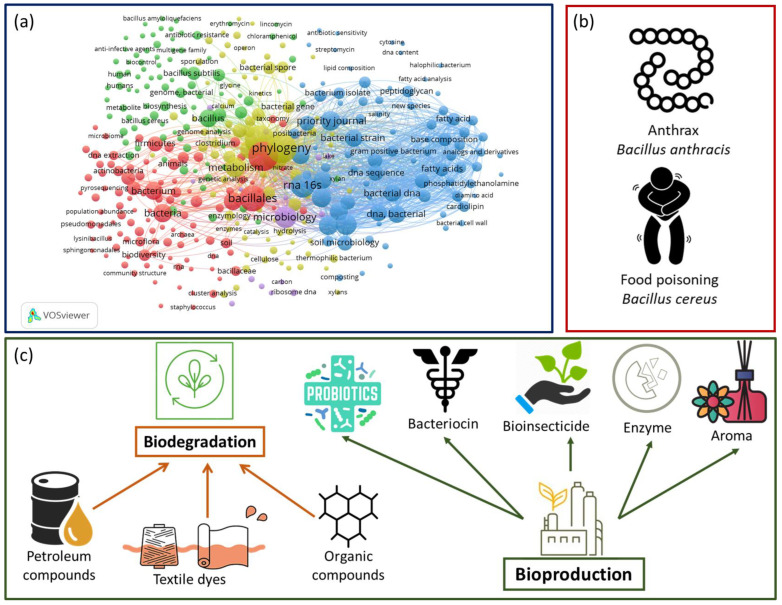
(**a**) The bibliometric map of *Bacillales* from 2000 to present, (**b**) their negative aspects on health, and (**c**) their positive aspects in the biodegradation and biorefinery applications.

The current review provides an inclusive overview of the order *Bacillales*, covering updates to the taxonomy of the order, its general features, diversity, and relevant industrial applications. *Bacillales*, which can be isolated mainly from the soil, have been the core of various research in the last 20 years, from their molecular characterization to bioengineered studies (Figure 1a). Among their species, *Bacillus anthracis*, which produces anthrax, and *Bacillus cereus*, which can cause food poisoning, can be given as an example of the negative aspects of Bacillales (Figure 1b). On the other hand, as discussed in further sections in this review article, their positive aspects have become more attractive in the industry since *Bacillales* play an active role in both biodegradation of petroleum-derived compounds, textile dyes, and aromatic hydrocarbons and the production of bioproducts such as organic acids, chemicals, surfactants, enzymes, and insecticides. (Figure 1c).

This review does not discuss, in detail, the pathogenic strains of the order *Bacillales*, though readers can refer to Gherardi et al. [4], Little and Ivins [5], Ehling-Schulz et al. [6], Hansen et al. [7], and Madigan et al. [8] on these topics.

Herein, we provided an overview of the order *Bacillales* with the recent updates in the number of families and genera allocated to this order and elucidated its taxonomical complications. As the genus *Bacillus* plays essential roles in the various industrial fields, we considered it separately due to its clinical significance. Moreover, the present review focused on new and less-known genera, providing a good background for their biotechnological potential. In the concluding part of the review, we introduced some examples of engineered strains belonging to the various species of the order *Bacillales*.

## 2. Order Bacillales

### 2.1. Taxonomy

In the revised roadmap of the phylum *Firmicutes*, Ludwig et al. 2009 delineated the *Bacillales* Prévot 1953 (Approved Lists 1980), the type order of the class *Bacilli*, to include eight families [9,10]. This followed the removal of two families, including *Caryophanaceae* Peshkoff 1939 (Approved Lists 1980) and the reassignment of its type genus, *Caryophanon*, to *Planococcaceae* Krasilnikov 1949 (Approved Lists 1980), despite the priority of the former family [9,11]. Of note, the authors also questioned the descriptive validity of the name *Caryophanaceae*, thereby virtually promoting the propagation of the name *Bacillales* Prévot 1953 (Approved Lists 1980). The Approved Lists 1980 included the above families and the orders *Caryophanales* Peshkoff 1939 and *Bacillales* Prévot 1953 [11]. As highlighted recently by Tindall 2019, the list did not provide a clear recommendation for the assignment of taxa above the genus level and, since only one name could correctly represent the order, considering appropriate rules of the International Code of Nomenclature of Prokaryotes. Considering the priority of *Caryophanales* Peshkoff 1939 over *Bacillales* Prévot 1953 and the inclusion of the nomenclatural types of both, *Caryophanon* Peshkoff 1939 and *Bacillus* Cohn 1872, respectively, in the same order, the correct name of the order is *Caryophanales* Peshkoff 1939 [12,13]. Thus, *Bacillales* Prévot 1953 (Approved Lists 1980) represents the later heterotypic synonyms of *Caryophanales* Peshkoff 1939 [11,13]. Having clarified the taxonomy, we are inclined to use the name *Bacillales* in the current review as the community gets accustomed to the correct name, *Caryophanales*.

The Order *Bacillales*, the type order of the class *Bacilli*, was approved in the list of bacterial names in 1980 [2,14]. Bergey’s Manual of Systematic Bacteriology has completely described this order completely based on 16S rRNA gene sequencing analysis and other polyphasic approaches such as chemotaxonomy and phenotypic methods [2,15]. Recently, based on the rules provided by The International Code of Nomenclature of Prokaryotes (ICNP), the name *Caryophanales* should be used instead of the name *Bacillales*. Since these names are heterotypic synonyms, however, the name approved first has the priority to be used. In this case, the name *Caryophanales* Peshkoff 1933 was approved prior to the name *Bacillales* Prévot 1953. Hence, the name *Caryophanales* Peshkoff 1933 is the correct name for the order *Bacillales* [13]. As the name *Caryophanales* is rarely used, and due to the earlier publication of the genus name *Bacillus*, the name *Bacillales* would be preferred to be used in this review.

As with other taxa, the previous classification of the order *Bacillales* relied heavily on 16S rRNA gene sequences, resulting in noticeable anomalies. For example, several spores- and non-spore-forming families and genera group together, suggesting that a single gene marker does not provide sufficient resolving power for delineating the order *Bacillales* [16]. Recent phylogenomic approaches, notably the work of de Maayer et al. 2019, attempted to resolve the evolutionary relationship among strains affiliated to the order based on comparative genomics [16,17].

Despite referencing the later heterotypic synonym, *Bacillales*, the authors proposed eleven distinct families in the order *Bacillales* and an unplaced group (Table 1).

The allocation of the Incertae sedis families agrees with the phylogenetic outline in volume 3 of Bergey’s Manual of Systematic Bacteriology [2,14]. Although *Bacillaceae* is the most imposing and well-known family, *Listeriaceae* consisting of *Listeria* and *Brochothrix* is also noteworthy. Moreover, *Pasteuriaceae*, some genera of the family *Paenibacillaceae* such as *Gorillibacterium* or *Brevibacillus*, and *Staphylococcus* have clinical and pathological importance [2,8]. Based on a consensus phylogenomic strategy, de Maayer et al. resolved the observed anomalies in the family *Bacillaceae*. For instance, although the genus *Staphylococcus* comprises pathogenic strains previously grouped in the family *Micrococcaceae*, the phylogenetic and molecular analysis did not reveal any close relationships between them, resulting in the proposal for a new family *Staphylococcaceae* [16]. However, the precise identification of its members at the species level needs arduous efforts and may fail if based only on phenotypic approaches [4]. Another heterogeneous and polyphyletic family in this order is *Planococcaceae*, with many phylogenetic works focused on the history of its evolution, but its demarcation remains unresolved [16,18,19]. The classification in this family is based on 16S rRNA gene nucleotide signature, phenotypic characteristics, and observed branching in the drawn phylogenetic trees. However, these methods do not have enough resolving power at the genus and species level and cause significant overlap with other species of the families *Bacillaceae* and Incertae sedis 19 [16]. It is noteworthy that *Planococcaceae* represents the later heterotypic synonym of *Caryophanaceae*, as highlighted earlier by Tindal [13] and recently supported by Gupta and Patal [20].

Below the family level, the 16s rRNA gene-based classification of the genus *Bacillus* and especially the Cereus clade also represents some challenges [21]. This method remains the gold standard for assigning microbial strains to various taxa due to its low-cost and reproducibility between laboratories worldwide, providing us with an overview of the microbial strains for further research. At present, the Cereus clade comprises *B. cereus*, *B. anthracis*, *Bacillus thuringiensis*, *Bacillus mycoides*, *Bacillus pseudomycoides*, *Bacillus cytotoxicus*, and *Bacillus toyonensis*. Different phylogenomics approaches have clarified the interrelationship of the otherwise incoherent genus *Bacillus* [6,21,22]. Gupta et al. 2020 also showed that a subset of the core proteins of *Bacillus* species, including concatenated proteins GyrA_B-RpoB-C and PolA-UvrD, provide consistent clustering strain in the genus [4,23]. In addition to whole genome approaches, which provide consistent means of identifying and classifying *Bacillales* at different taxonomic ranks, relevant techniques for identifying industrially important strains may be pertinent toward effective industrial deployment [16,24,25].

**Table 1:** List of families assigned to the order *Bacillales* [2,13,14,16,20,22,26,27].

Family Name	Proposed by	IJSEM ^1^ Validation List No. (Year)	Type Genus	Involved Valid and Invalid Genera Until 2022 in LPSN ^2^	Valid Genera with Correct Name until 2022 in LPSN
*Alicyclobacillaceae*	da Costa and Rainey 2010	132 (2010)	*Alicyclobacillus*	5	5
*Bacillaceae*	Fischer 1895	(1980)	*Bacillus*	134	117
*Caryophanaceae*	Peshkoff 1939	(1980)	*Caryophanon*	26	19
*Desulfuribacillaceae*	Sorokin et al. 2021	200 (2021)	*Desulfuribacillus*	1	1
*Listeriaceae*	Ludwig et al. 2010	132 (2010)	*Listeria*	3	2
*Paenibacillaceae*	De Vos et al. 2010	132 (2010)	*Paenibacillus*	19	16
*Pasteuriaceae*	Laurent 1890	(1980)	*Pasteuria*	1	1
*Sporolactobacillaceae*	Ludwig et al. 2010	132 (2010)	*Sporolactobacillus*	5	5
*Staphylococcaceae*	Schleifer and Bell 2010	132 (2010)	*Staphylococcus*	12	10
*Thermoactinomycetaceae*	Matsuo et al. 2006	(2007)	*Thermoactinomyces*	25	24
Incertae sedis 46	-	-	-	1	1

^1^ International Journal of Systematic and Evolutionary Microbiology. ^2^ List of Prokaryotic names with Standing in Nomenclature.

### 2.2. General Characteristics

General characteristics of bacteria include a wide range of features such as the shape and arrangement of the bacterial cells, cell wall chemical type, spore formation ability, motility, growth conditions, and resistance and tolerance to antibiotics. [8,28]. Interestingly, the genera of order *Bacillales* are phenotypically diverse. For example, cell shapes change from spherical to filamentous and may be motile by flagella. Oxygen requirement has a broad range from strictly aerobic, microaerophilic, facultative anaerobic, and aerotolerant to strictly anaerobic [2,29]. One of the most important characteristics of the order *Bacillales* is endospore formation, although some exceptions exist [2,3]. However, these exceptions remained to be clarified whether from one side lacking the genes for spore formation, or losing all or some of them during evolution, or alternatively, unfavorable physiological conditions cause unsuccessful spore formation [17]. Furthermore, the cell wall of order *Bacillales* stains Gram-positive for young cells, in general, but some genera react Gram-negatively, such as *Aidingibacillus*, *Aquisalibacillus*, *Bhargavaea*, *Caldibacillus*, *Caryophanon*, *Exiguobacterium*, *Mammaliicoccus*, *Novibacillus*, *Sediminibacillus*, and *Thalassorhabdus* [2,14,29,30]. Additionally, most of the genera allocated to this order have menaquinone 7 (MK-7) as their respiratory quinone, while various exceptions have been found [2].

## 3. Genus Bacillus

### 3.1. Genotypic Characteristics

In the taxonomic outline of phylum *Firmicutes* and the order *Bacillales*, the family *Bacillaceae* contains 117 validly published genera, of which the type genus is *Bacillus*. For the first time, in 1872, Ferdinand Cohn, a German bacteriologist, described Gram-positive endospore-forming rods and, therefore, named them *Bacillus* [2,22]. Afterward, many types of research have been carried out on this genus providing valuable information about it and revealing various potentials and new features of the genus *Bacillus*. However, new investigations are currently being conducted around the world to clarify the evolutionary history of this divergent genus [22,26]. Based on phylogenetic studies, there are various clades consisting of several species within the genus *Bacillus* [2,14,31]. Particularly, 16S rRNA gene sequencing analysis is not effective lonely to differentiate species or strains within species, and other approaches, such as DNA/DNA hybridization (DDH), whole-cell proteins profile, RFLP, multilocus sequence typing (MLST) using housekeeping genes such as *glpF* (glycerol uptake facilitator protein), *gyrB* (DNA gyrase subunit B), *ilvD* (dihydroxy-acid dehydratase), *gmk* (guanylate kinase, putative), *pycA* (pyruvate carboxylase), *pta* (phosphate acetyltransferase), *pur* (phosphoribosylaminoimidazolecarboxamide), *tpi* (triosephosphate isomerase), and multilocus enzyme electrophoresis (MLEE) or average nucleotide identity based on BLAST (ANIb), should be tested for precise results [2,23,32,33,34,35]. In general, polyphyly and excessive heterogeneity of species in the genus *Bacillus* are due to delicate criteria used in the last decades to allocate numerous species with different phenotypic properties to this genus. Therefore, the genus *Bacillus* shows great diversity as some species do not even have a common evolutionary history with the type species (*Bacillus subtilis*). In recent years, many species were reclassified, and novel genera such as *Gracilibacillus*, *Virgibacillus*, *Solibacillus*, *Ureibacillus*, and *Alicyclobacillus* were produced [22]. Up to now, genomes of 225 species of the genus *Bacillus* in National Center for Biotechnology Information (NCBI) genome database have been sequenced; however, genome sequencing and comparative genomic analysis can provide the opportunity to check evolutionary relationships of species making it possible to identify molecular markers (molecular synapomorphies) [22,36]. Molecular synapomorphies, which comprise conserved signature inserts and deletions (CSIs) in protein sequences, are consistent means for differentiating species from two main clades of the genus *Bacillus*, i.e., ‘Subtilis clade’ and ‘Cereus Clade’. Based on Rule 56a of the ICNP, species transfer from the Cereus clade into a novel genus may threaten human health; hence, it is not a logical and advisable transfer. However, all other species of the genus *Bacillus* not belonging to these clades should be transferred to new genera by means of reliable identification approaches [37]. According to evidence obtained from comparative genomic analysis of *Bacillaceae* species and 36 novel CSIs (Three unique CSIs from each clade of Firmus, Jeotgali, and Simplex; six CSIs from Fastidiosus clade; 10 CSIs from Alcalophilus clade; and 11 CSIs from Niacini clade), 103 misclassified and unrelated species of the genus *Bacillus* were assigned and moved to novel proposed species. Moreover, phylogenomic analysis was carried out on protein datasets such as core proteins and conserved proteins in the phylum Firmicutes. Additionally, concatenated sequences of highly conserved proteins such as PolA, RpoB, RpoC, GyrA, GyrB, and UvrD were analyzed. Interestingly, monophyletic groups observed in all reconstructed phylogenetic trees were named Alcalophilus, Fastidiosus, Firmus, Jeotgali, Niacini, and Simplex clades and confirmed through comprehensive comparative genomic analysis of aforementioned protein sequences [22].

### 3.2. Phenotypic, Chemotaxonomic, and Morphological Characteristics

Based on morphological studies, cells of the genus *Bacillus* are straight or slightly curved, Gram-positive, or even Gram-negative rod-shaped with round-ended except Cereus clade members that have squared-ended cells, singly or in pairs, sometimes chains, or long filaments that can produce very variable colonies from raised to convex, small to large, circular to the irregular shape and smooth to rough texture with entire to fimbriate edges under different growth conditions but the recognition of *Bacillus* colonies in the routine growth media such as nutrient agar or tryptic soy agar is not too difficult. In addition, it should be noted that if the staining slide is prepared from the old culture, it would be stained Gram-negatively and may cause a misunderstanding of the precise Gram type of the cell wall. Normally, the most common cross-linkage in the cell wall of the genus *Bacillus* is *meso*-diaminopimelic acid (*meso*-DAP) direct murein, but infrequently, other types of cross-linkage, including l-Lys-d-Glu, Orn-d-Glu, and l-Orn-d-Asp, have been reported. The cytoplasm of cells can be observed as an area less refractive than a spore by phase-contrast microscopy. This vacuolated area is due to storage materials that are produced in the presence of fermentable carbohydrates such as glucose [2,28].

*Bacillus* species can form only one endospore in a cell, and many factors like manganese ions, the depletion of nutritional elements in the medium, cell density, desiccation, or the fluctuation in pH or temperature can induce sporulation [3,38,39]. Spore formation is one of the most valuable characteristics used to identify *Bacillus* species. The genus *Bacillus* has widespread habitats as its species can be isolated mostly from soils or other environmental sources, including air, sediments, sludges, fresh or marine waters, hot springs (acidophiles and thermophiles), soda lakes (alkaliphiles), hypersaline lakes, or salterns (halophiles), clinical specimens, foods, wastes, composts or manures, animals appendages like feathers, invertebrates, stone surface, clean rooms, and wall paintings. This diversity impressively shows that the members of this genus can be active metabolically in various environments, but it needs more research to find out all of the interactions taking place between them and other microorganisms living together [2,3,35,40].

*Bacillus* growth requirement (one of the most considerable characteristics for the identification of species) demonstrates very wild diversity within the genus, but these are not so complicated; also, most species can grow heterotrophically on simple or enriched media such as nutrient agar or blood agar, respectively. In addition, the *Bacillus* genus can utilize or assimilate carbohydrates unless for the mentioned exceptions, such as *Bacillus benzoevorans* which requires benzoate or acetate salts for its growth [41]. Furthermore, some species need special conditions for growth, e.g., alkaliphiles that love alkalinity environments or halophilic ones that need sodium ions for their growth [42]. Most species do not need growth factors like vitamins; however, yeast extract can be useful to stimulate growth. In general, *Bacillus* species are chemoorganotrophic, but some strains can exhibit chemolithotrophic growth [2].

Another general characteristic of *Bacillus* is the growth temperature range from low (−2–20 °C) (psychrophiles) to very high (>50–70 °C) (thermophiles) temperatures, but most of the species prefer to grow under mesophilic conditions (25–40 °C and usually 30 °C). Oxygen requirement varies from aerobic, facultative anaerobic to strictly anaerobic within the genus so that most species can produce catalase, while some of them can produce oxidase. Despite the high diversity of *Bacillus* species, most of them are not recognized as pathogenic agents in humans or animals. However, there are some exceptions, the most considerable of which is *B. anthracis* causing anthrax. However, a few species cause food poisoning, and some of them may act as opportunistic agents in cases with immune deficiency [2,29].

Several general characteristics have been mentioned for the identification of the genus *Bacillus*, but not all of them play any significant role in the taxonomy of this genus. For example, L-form cells that have lost their cell wall as the stable type cannot return to the original shape, and the spheroplasts can revert to the original bacterial form in the genus *Bacillus*. Thus, this is not a crucial characteristic in taxonomical studies [2,43]. Moreover, another example refers to the capsule -a sticky layer around the cell. Its types are discussed among *Bacillus* species so that some species produce polysaccharide capsules, and others produce poly-γ-d-glutamic acid capsules, while several species, such as *Bacillus megaterium* (now *Priestia megaterium*), can form both types. Capsule looks like a double-edged structure that can be useful in the soybean fermentation process or act as a virulence factor to help the colonization of pathogenic species in a host [2,28]. In addition to the capsule, some species of the genus *Bacillus* can form two-dimensional stable and porous surface layers. These layers are composed of protein self-assembly or glycoprotein molecules. S-layer is not a valuable marker in the taxonomy, and often its presence is strain-dependent [2,44]. Motility is another character that is not considered an appropriate tool for the taxonomy of this genus. Though, it is typically used to describe most species [45]. Many techniques are applied to improve bacterial taxonomy, including fatty acids analysis, whole-cell proteins profile, phage-typing, and serotyping, so these may be essential to differentiate strains of a species [2,8].

### 3.3. Pathological Importance

A few species of the genus *Bacillus* are related to diseases in humans or animals, including invertebrates. The main pathogenic species is *B. anthracis*, which has been categorized as a bioterrorism agent. Principally, this species causes a bacterial infection in herbivorous animals, and before the 1930s, it was the most important causative agent in the mortality of livestock such as horses, cattle, or sheep [46,47]. Development and progress in vaccination have decreased the incidence rate of anthrax. However, this disease is marked as endemic in many countries in Asia, Africa, and even Europe. *B. anthracis* cannot be eradicated because of the long-lasting existence of its spore in the ecosystems like soils. Anthrax in humans includes cutaneous, intestinal, and inhalational infections, among which the cutaneous is the most common form and the latter is the more lethal form. Since it is not recognizable in the initial steps, it would be too difficult to do appropriate treatments. In this species, two plasmids are responsible for the pathogenicity and virulence factor named pXO1 and pXO2, respectively. The pXO1 encodes the toxin complex, including three modules, protective antigen (PA), edema factor (EF), and lethal factor (LF), none of which are individually toxic. The pXO2 contains capsule genes that are responsible for the production of a proteinaceous polymer. It consists of γ-d-glutamic acid with a negative charge as its monomeric subunits and plays a protective role in struggling phagocytosis in the host. However, by conducting the plasmid curing in the cells, they will be efficiently converted into avirulent strains [5,6].

*B. cereus* is already known as the second important toxigenic species of the genus *Bacillus* that causes food poisoning syndromes—diarrhoeal and emetic types—or even opportunistic sicknesses because of its spores. They can tolerate normal cooking processes and acidic conditions of the stomach to survive and produce toxins in the small intestine. This species can be easily found in meats, eggs, and herbal foods like fried rice or dairy products. Additionally, several strains of this species can be infectious in the animals like cattle [2,35]. However, there are some reports about the strains that can be applied for biotechnological applications, e.g., fungicidal materials produced by *B. cereus* UW85 (34). In addition, *B. thuringiensis*, one of the Cereus clade members, is an invertebrates’ pathogen typically used as a biocontrol agent. This species produces an insecticidal crystalline in its spore that is structurally proteinaceous endotoxin and encoded by the conjugative plasmids affecting insects such as butterflies, beetles, moths, flies, or even some of the nematodes, protozoa, mites, and flatworms [2,48,49,50]. As there are more than 80 types of this protein, its nomenclature is not easily defined; therefore, a committee was established in 1993 to update the old nomenclature based on amino acid identity. For further information about the newly constructed databases, the readers are referred to visit http://www.lifesci.sussex.ac.uk/home/Neil_Crickmore/Bt/intro.html, accessed on 22 November 2022. There are a few reports about the pathogenicity of the other species within the genus *Bacillus*. Molecular mechanisms of pathogenicity and further information about these mentioned species have already been discussed in numerous articles and books; therefore, they will not be at the center of our attention in this review.

### 3.4. Sporulation and Spore Properties

The ability of microorganisms that produce resistant forms is to survive in adverse conditions that can be lethal for the vegetative cells. Various research about spores has been conducted, but there are still unknown details about their molecular mechanisms, genetics, and resistance properties [51,52]. A spore is an optically refractive body whose shape can be ranged from cylindrical, ellipsoidal, spherical, or irregular forms in the genus *Bacillus*. Moreover, the position of the spore within the cell is important and can be used in the identification of the species. Spores may be located in the central, paracentral, subterminal, or terminal positions of the cells. However, within a species, often, strains exhibit some variations in the shape or even in the position of spores. Depending on the sporangia size, it can be swollen, but non-swollen sporangia are common in the genus *Bacillus* [2].

Sporulation is a sophisticated process that causes spore formation—the most durable form of life on the earth—during a few hours. Both internal and external signals affect the cell to make a decision for the initiation of the sporulation. In exposure to sufficient amounts of water and nutrients such as sugars or amino acids, especially L-alanine, and under favorable conditions, germination of the spore will occur, and new vegetative cells will emerge. Spore formation has been studied completely in *B. subtilis* strain 168 as a model and contains several stages that commence with the phosphorylation of an important transcriptional regulatory protein named Spo0A [38,39,53]. Spo0A is a master regulator which controls the expression of more than 120 genes directly and can affect the expression of over 500 genes interfering in the sporulation. In addition to Spo0A, the accurate and correct function of specific sigma factors ensure successive process [38]. At the beginning stage, the vegetative cell undergoes an asymmetric cell division, and an axial filament of the cell chromosome forms. A specific area of the elongated chromosome migrates toward one of the cell poles, and then the forespore configures gradually. One the bases of the spore formation model, this process can consist of seven stages: 1, pre-septation or axial filament formation; 2, asymmetric division; 3, forespore formation; 4, spore cortex formation; 5, synthesis of spore coats proteins and their deposition out of the cortex; 6, maturation of spore; 7, lysis of the sporangium and spore releasing [2]. Although morphological aspects of this process are the same among aerobic endospore-forming bacteria, there might be some differences between genera. The spore is metabolically inactive with a complex ultrastructural core. It involves DNA, RNA, proteins, dipicolinic acid (pyridine-2,6-dicarboxylic acid, DPA), and calcium ions surrounded by various layers including inner membrane, cell wall, cortex, outer membrane, inner spore coats, outer spore coats, and exosporium from the innermost to outermost, respectively [54]. These layers are responsible for the protection of spores against solvents and chemicals, degradative enzymes, desiccation, thermal shocks, ultraviolet (UV) radiation, and other stresses [2,51,55]. It is hard to say what is the exact mechanism of spore resistance to these stresses, but it is also assumed small acid-soluble proteins (SASPs) play a significant role in protecting the chromosomal part of the spore [51]. A spore is a resistant form of life in endospore-forming bacteria. Therefore, these kinds of bacteria are in the spotlight of research for industrial applications. Moreover, *Bacillus* spores are used in spore surface display technology and many other applications, such as metal bioremediation (17) or biosensors [56]. Later, we will point out in detail about this group of bacteria, particularly endospore-forming genera of the order *Bacillales*.

### 3.5. Industrial Applications

Nowadays, the genus *Bacillus* is variously used for industrial purposes and can be recognized as generally recognized as safe (GRAS), i.e., non-toxicogenic and non-pathogenic. What makes this genus one of the most potent microorganisms in the industry involves metabolic diversity, precise understanding of genetics and physiology, non-pathogenicity except for a few species, fast growth rates, high cell density, noteworthy resistance to harsh conditions, and production of various metabolites such as enzymes, amino acids, vitamins, surfactants, and bioactive compounds [29,55,57,58,59,60,61]. At present, the usage of enzymes is not only limited to laboratories or research, but also, they are used in our homes. Enzymes of this genus occupy approximately 50% of the total marketplace, and the best commercial suppliers are Novo Nordisk (Denmark) and Genencor International (USA), but many other companies throughout the world are supplying *Bacillus* enzymes [55]. The majority of *Bacillus*-produced enzymes are extracellular; however, this genus can produce substantial intracellular enzymes such as glucose iso-merase, as discussed in Section 3.5.1, glycerokinase [62], glucokinase [63], leucine dehydrogenase [64,65], superoxide dismutase [66], and Rhodanese [67]. Table 2 shows the most important enzymes produced by various *Bacillus* spp. and their applications. Among these species, *Bacillus licheniformis* is a remarkable species for the production of extracellular enzymes such as β-lactamase, thermostable α-amylase, and protease in large scales [68]. Having considered the different abilities and usages in the industry, we summarized the genus *Bacillus* applications in several parts followed.

#### 3.5.1. Food Industry

For a long time, microorganisms have been used in the food industry, mainly in dairy and fermented products. One of the most commercially significant species of this genus is *B. subtilis* which can secret produced enzymes into the media. It is also applied on a large scale to make natto (itihiki-natto) from soybeans, a traditional Japanese fermented food [55]. In 2008, *B. subtilis* was approved by European Food Safety Authority as a qualified presumption of safety (QPS). Moreover, it can be used as a GRAS host for the production of recombinant compounds as He et al. [27,57,69,70] constructed a food-grade recombinant *B. subtilis* cable to transform D-fructose to D-allulose, an uncommon sugar for the substitution of sucrose in the food industry. Another GRAS species is *Bacillus amyloliquefaciens*, the most imperative species for the worldwide production of hydrolytic enzymes. Its β-glucanases can be used in the wine industry to reduce the maturation process. Moreover, its metalloproteases have been used to prevent protein fogs in beer or to decrease the gluten content in cookies or biscuits flours. Iso-amylase and α-amylase produced by this species are abundantly used for the syrups preparation, liquefaction of starch, corn starch, brewing industry, and many other applications, such as textile and paper industries [28,51,69,71]. *B. amyloliquefaciens* was isolated in 1943 but approved in 1987 based on rules 24a and 28a of the International Code of Nomenclature of Bacteria (ICNB) [69,72].

In addition, *Bacillus polymyxa* CECT 155 (now *Paenibacillus polymyxa*), *Bacillus* sp. US 149, *Bacillus* sp. KSM-1876, *Bacillus* sp. KSM 1378, *Fontibacillus* sp. strain DSHK from the family *Paenibacillaceae*, and *Pullulanibacillus naganoensis* strain AE-PL from the family *Sporolactobacillaceae* can hydrolyze different oligo- or polysaccharides by producing a variety of debranching enzymes of the family pullulanase that break α- 1,6 linkages. Latter production has been used in the food industry [73,74]. Generally, these enzymes are used to manufacture concentrated glucose or high maltose corn syrups used in the production of candy and ice cream, as the sweeteners in beverages, or as the improving agents in the bakery industry. Furthermore, this enzyme family, in combination with alkaline α-amylase, can be applied as a detergent in dishwashing or laundry to remove starchy spots or as lubricants, emulsifiers, thickening and gelling agents [73,75]. In addition to extracellular enzymes, D-xylose iso-merase (glucose iso-merase) produced by *Bacillus coagulans* (now *Weizmannia coagulans*) is the earliest instance of an intracellular and immobilized enzyme used in the food industry. It is used to prepare the high fructose corn syrups wildly applied as sweeteners in diabetic foods because they do not need insulin to be metabolized in the body. This enzyme is very well-matched with industrial conditions as the optimal temperature and pH for its activity are 55–65 °C and 7–9, respectively, which are significant factors in starch hydrolyzing [29,55,75]. Presently, some *Bacillus* species are used for the fermentation and post-harvesting processes of foods and grains. For example, *P. megaterium* can produce short-chain fatty acids during the latest stage of cacao beans fermentation which, along with other chemicals, contributes to an off-flavored smell [76]. *Bacillus circulans* (now *Niallia circulans*), in addition to *B. amyloliquefaciens,* is another commercial producer of β-glucanases [29].

Along with enzymes, *Bacillus* species can produce various bioactive compounds such as antiviral and antitumor agents, antimicrobial small peptides (AMPs), or bacteriocins such as tyrocidine, gramicidin, polymyxin, amylolysin, and bacitracin, the latter of which inhibits the growth of *Streptococcus pyogenes* proficiently [77,78]. These species include *B. licheniformis* [79], *B. mycoides* [80], *Bacillus halodurans* (now *Halalkalibacterium halodurans*), *Bacillus mojavensis* [81], *Bacillus pumilus* WAPB4 [82], *Bacillus sonorensis* MT93 [83], *Bacillus* sp. P11 [84], *B. amyloliquefaciens* [85], and *B. subtilis* [36]. Antimicrobial small peptides can be used as food preservatives to improve the quality of foods and prevent spoilage [59]. For instance, cerein 8A has been applied in soft cheese and milk to control the growth of *Listeria monocytogenes* [86]. AMPs-producers are in the spotlight of the pharmaceutical industry and medicine, which will discuss in the following section.

*Bacillus* whole cells and spores are broadly used as probiotics (Generally live microorganisms that provide health benefits when eaten in acceptable dosage) in human foods, supplements, livestock feed, and aquafarming to promote the growth of aquatic organisms and decrease the chemical food additives applications as much as possible [57,87,88]. The best examples of *Bacillus* probiotics are *Bacillus clausii* (now *Shouchella clausii*), *W. coagulans*, *B. subtilis*, *B. licheniformis*, and *B. cereus*. Spore formation in this genus makes it a convenient choice compared to other probiotics like *Lactobacillus* or yeasts, which do not form a spore. Therefore, it is possible to store them at ambient temperature without any problems. Consequently, it reduces preservation and shelf costs. It is shown that *Bacillus* probiotics can stimulate the immune system and have antimicrobial activities due to their bioproducts. Nevertheless, microorganisms and their products should be considered under regulatory roles and assessments such as QPS to ensure consumers. Moreover, it should be verified that these microorganisms and products do not threaten human and other organisms’ life via toxigenic and antibiotic resistance genes [36,87,89].

#### 3.5.2. Pharmaceutical Industry

The current pharmaceutical industry emerged in the late nineteenth century but studying the history indicates our ancestors were able to find curative herbal drugs from nature based on trial and error [90,91]. In this industry, microorganisms are substantial sources of growth hormones, insulin, monoclonal antibodies (mAb), or recombinant interferon production. Whatever has led pharmaceutical companies to change their upstream strategies from animal-based to cell-factory-based production is associated with hypersensitivity of the human body to animal serum and the contamination risk of biopharmaceuticals with prions [92]. *Escherichia coli* is the most universally used host to produce recombinant proteins, though *B. subtilis* is an adequate alternative to be used frequently as a host for sustainable products. It can affect the downstream processing of biopharmaceuticals [55,93]. The lack of an outer membrane containing lipopolysaccharides (LPS) is an important property that convinced the pharmaceutical industry to use the genus *Bacillus* instead of Gram-negative bacteria. In general, LPS are stated as endotoxins interfering purification of final products. Moreover, the secretion capacity of this genus has made it more attractive than *E. coli* since this ability may result in the natural exudation of the products into the media, which, in turn, can facilitate downstream processing [94]. However, it should be noted that there are few reports regarding the production of human recombinant proteins in the genus *Bacillus* as a host. Influential reasons that restricted *Bacillus* usage for the production of these proteins relate to the lack of applicable expression systems, instability of plasmids, degradation of produced proteins by proteases, and protein misfolding. However, numerous empirical solutions have been found to overcome these problems due to a wide range of research, such as omics approaches and metabolic engineering, conducted accordingly [94,95,96].

Undeniably, the discovery of antibiotics has been effective in revolutionizing medicine and treating infectious diseases. In general, antibiotics are one of the significant pharmaceutical products produced by microorganisms or synthetically by man. In addition to fungi, the genus *Bacillus* can produce four classes of antibiotics; cyclic or liner small oligopeptides, basic peptides, and aminoglycoside compounds [77]. These antibiotics can be classified based on synthesis mechanisms and ribosomal or non-ribosomal pathways [97]. Many species like *B. subtilis* produce more than one type of bacteriocins, such as subtilin, sublancin 168, iturin, mycobacillin, fengycin, and pumilacidin, or surfactin with surfactant activity [98,99]. Surfactin is a robust, low-toxic and biodegradable lipopeptide biosurfactant synthesized non-ribosomally by a synthetase complex. Its properties make it suitable to be used in many industries, particularly in oil recovery. In the presence of this biomolecule, the wettability index of a system can be changed because of a decrease in the interfacial tension between organic materials and water interfaces [36,100,101,102].

Another important bacteriocin is bacitracin, the first AMP discovered in *B. licheniformis* culture, which is broadly used in human and veterinary medicines [103,104]. In addition to the antibacterial activity of AMPs, these compounds showed anticancer or antifungal activities [105]. However, the new generation of antibiotics should be effective against drug-resistant pathogens such as methicillin-resistant *Staphylococcus aureus* (MRSA), vancomycin-resistant *E. coli*, *Pseudomonas aeruginosa*, or *Acinetobacter baumannii* that are very serious causative agents in nosocomial infections and treat public health. Nevertheless, what is essential to control these resistant strains relates to researchers’ attempt to exploit such abilities (broad-spectrum, fast killing, and high efficacy) among *Bacillus*-produced AMPs by using cutting-edge approaches, isolating new strains from unusual environments or making modified types of them using bioengineering [106]. For therapeutic applications in humans, it should not be neglected to study their mechanism of action, immunogenicity, and toxicity before clinical trials. Another substantial potential of *Bacillus* species, especially *B. cereus,* in the pharmaceutical industry is related to their ability to produce β-lactamases (Type I and II). These enzymes are responsible for the resistance to β-lactam antibiotics where the infected patients cannot be recovered and need to be treated with non-β-lactam antibiotics. Beyond the clinical importance of β-lactamases, these enzymes can be used in the microbiological assay, sterility testing in the antibiotic industry, or susceptibility assay of novel antibiotics to β-lactamases [29].

**Table 2:** Most important enzymes produced by various *Bacillus* spp.

Species Name	Enzyme Class	Main Industrial Application	Reference
*B. amyloliquefaciens* *B. subtilis* *B. thuringiensis*	Extracellular metalloprotease	Leather industry	[107,108,109]
*B. amyloliquefaciens* *B. licheniformis* *B. subtilis* *B. pumilus*	Serine protease	Detergent industry Antifungal agent	[110,111,112,113]
*B. alcalophilus* *B. amyloliquefaciens* *B. licheniformis* *B. subtilis* *B. thuringiensis*	α-Amylase	Starch and food industries	[114,115,116,117,118]
*B. cereus* var. *mycoides*	β-Amylase	Starch industry	[119]
*B. cereus* FDTA-13 Thermophilic *Bacillus* sp. AN-7	pullulanase		[120,121]
*Bacillus* sp. CICIM 304	Iso-amylase		[122]
*Bacillus* sp. CSB55 *B. subtilis*	β-glucanase		[123,124]
*B. subtilis* *B. licheniformis*	β-lactamase (Penicillinase)	Molecular Biology Antibiotic industry	[125,126]
*B. licheniformis*	Glucose iso-merase	Food industry	[127]
*Bacillus methylotrophicus* *B. subtilis*	Levansucrase	Food and pharmaceutical industries	[128,129]
*B. subtilis* *B. pseudomycoides* *B. licheniformis*	Cellulase	Biofuel industry	[130,131,132]
*Bacillus australimaris* *Bacillus tequilensis*	Xylanase	Biofuel and paper industries	[133,134]
*B. licheniformis* *Bacillus* sp. G-825-6 *Bacillus* sp. ND1	Cyclodextrin glucanotransferase (CGTase)	Pharmaceutical, food and cosmetic industries	[135,136,137]
*B. subtilis* E9 *B. mojavensis* TH309	Esterase	Biodegradation	[138,139]
*B. thuringiensis* *B. licheniformis* B307	Chitinase	Biocontrol agent	[140,141]
*B. velezensis* TA3 *B.cereus* *B. subtilis*	Tannase	Delignification process	[142,143,144]

#### 3.5.3. Agricultural Industry

It is quite obvious that *B. thuringiensis*—discovered by Shigetane Ishiwatari in 1902—is the most related species in the agricultural industry. As discussed earlier, this species has been considered a biocontrol agent for invertebrates, especially insects, due to its spore endotoxins and secondary metabolites [59,145]. Its sporangium is identified by parasporal inclusions of crystalline *Bt* endotoxins. These kinds of inclusions can be produced by *Lysinibacillus sphaericus,* which is toxic for mosquito larvae. Therefore, this should be noted to avoid confusion in the identification process of these species [35,146]. Some *Bt* toxins (Vip toxins) can be produced in vegetative cells, but the main production phase is during sporulation [147]. In addition to this toxic crystalline structure, *B. thuringiensis* produces phospholipase C, proteases, and hemolysins as virulence factors, but they are not adequate for a strain to be a pathogen [148,149]. Two types of *Bt* toxins have been identified, Cyt and Cry toxins [150]. Attempts to discover more insecticidal genes among toxigenic strains are continuing. For example, *cryI* gene encodes the toxic protein for lepidopterans, while *cryII* and *cryIII* genes encode toxins for dipterans and coleopterans, respectively [151]. One of the suggested action modes of *Bt* toxins (activated by insect midgut proteases) in the target insect is midgut epithelial cell death by forming ionic pores into the membrane and following osmotic lysis [152]. Another proposed mechanism refers to the activation of the Mg^2+^-dependent signal cascade pathway via the adhesion of toxin to the primary receptor. This pathway can cause swelling and nuclear ghosts, and, eventually, cell death [153].

In the agricultural industry, toxicogenic products of *B. thuringiensis* (viable spore and poisonous proteins) are formulated into a powder, granule, or liquid to be applied for the corps and farms [48]. As *Bt* toxins affect the pests selectively and do not accumulate in the environment, it is broadly preferred over chemical pesticides that have non-specific actions, causing an increase in pesticide-resistant pests and hazardous effects in the environment [154,155]. However, unremitting usage of *Bt* toxins as a biocontrol agent should be avoided to prevent the incidence of resistance and also cross-resistance to these toxins (it means an insect population that was resistant to a specific toxin shows resistance to a toxin that has not been exposed before) [156]. Resistance modes include attenuating toxin binding to the cell receptors, reducing protoxin solubilization, toxin degradation, or altering toxin processing. Another disadvantage of *Bt* toxins is related to a narrow spectrum of their activity, particularly in cloned toxins in contrast to chemical pesticides. Therefore, it is so important to find successful strategies to improve toxins activity and contiguous usage to control pests in the fields [157].

In addition to *B. thuringiensis*, there are many species related to the agricultural industry, like diazotrophic *Bacillus* spp., for fixing molecular nitrogen. In general, nitrogen fixation is an essential process that molecular N_2_ is converted into ammonia for the biosynthesis of life macromolecules. Nitrogen fixation has been demonstrated in *L. sphaericus*, *P. megaterium*, *B. cereus*, and *B. licheniformis* isolated from plants’ rhizosphere. Some other strains may be classified in the genus *Paenibacillus* [158]. At the same time, recent phylogenetic research restricted aerobic spore-forming bacteria with nitrogen fixation capability to the genus *Paenibacillus* [159]. Perhaps, nitrogen-fixing *Bacillus* spp. promotes plant growth through the production of hormones such as auxins (indole-3-acetic acid, IAA) and inceases accessibility of nutritional materials through phosphate solubilization or iron acquisition (by siderophores such as schizokinen, bacillibactin, and petrobactin) [3,160]. Moreover, these bacteria can quench ethylene production, interact with other rhizosphere microorganisms, increase nodulation in roots, and produce antibiotics or fungicidal compounds (produced by *Bacillus velezensis* RC 218, *Bacillus endophyticus* (now *Priestia endophytica*), *Bacillus insolitus* (now *Psychrobacillus insolitus*), *P. megaterium*, *B. subtilis*, *B. pumilus*, *B. amyloliquefaciens* FZB42, and *B. licheniformis*) to protect plants from phytopathogens [2,146,161]. One of these products, named zwittermicin A, is an unusual linear aminopolyol compound and prevents fungal disease in plants [162,163]. Commercial examples of these fungicides are known as Ballad Plus, Sonata, and Biobest [164]. Recently, by genomic data mining, the discovery of antimicrobial gene clusters in the *Bacillus* strains has increased and made a big picture of these new antimicrobial compounds and their structures and activities [165].

#### 3.5.4. Other Industrial Applications of the Genus Bacillus

Unquestionably, the genus *Bacillus* plays a significant role in well-known industries. Moreover, with the different capabilities, it can be applied to problems that may need to be solved biologically. At present, various environmental issues are attractive for environmentalists and researchers to find efficient strategies to rescue ecosystems, remediated soils, waters, and air, and protect them from further damage. One of these strategies is related to the use of microorganisms and their capacities for the degradation of pollutants or remediation of contaminated areas. In this context, there are many examples of efficient microorganisms. There are many *Bacillus* species with the ability to change inorganic compounds by respiration. For example, *Bacillus subterraneus* (now *Mesobacillus subterraneus*) and *Bacillus infernus* can carry out nitrate respiration and use iron III, MnO_2_, and nitrate as electron acceptors [2]. It seems these species have a substantial role in the biogeochemical cycling of nitrogen and carbon, sulfur, phosphorous, and manganese [36]. It should be noted that some species can absorb metals non-enzymatically and aggregate them on their surfaces. In addition to the oxidation and reduction of inorganic materials, this genus can degrade, transform or metabolize intricate organic compounds, catalyze reactions that cannot be performed chemically, and remediate contaminated environments [2,3]. Based on the genus *Bacillus*’ diverse abilities, the petroleum industry is attentive to applying its species to solve existing problems that have not been unraveled by abiotic methods. For example, *Bacillus* spp., like *Bacillus firmus* (now *Cytobacillus firmus*), has a strain H_2_O-1 that can produce antibiotics against sulfate-reducing bacteria (SRB) and can be an appropriate choice to prevent pipelines bio-corrosion and improve oil recovery in the underground oil reservoirs [146,166,167]. One novel application of *Bacillus* in the fuel industry is linked with ethanol production from concentrated date syrup by a new strain of *B. amyloliquefaciens*. It is reported that this strain can produce 0.35 g L^−1^ h^−1^ by fermenting glucose, sucrose, and fructose under the high osmotic pressure of concentrated date syrup. It seems that isolating new strains with unusual abilities is promising for their usage in the advanced biotechnological industry [168].

Fascinatingly, some species of the genus *Bacillus* isolated from marine or other sources can produce other secondary metabolites such as terpenes, antimicrobial fatty acids, and polyketides (PKs) in addition to AMPs. These secondary metabolites are synthesized by molecular complexes using proteins rather than nucleic acids as the templates, called polyketide synthases (PKSs). They are indeed grouped into three classes based on the genes which encoded proteins involved in the biosynthesis pathway: Type I (multi-domain and bulky proteins), II (separated proteins), and III [169,170]. Some examples of these compounds produced by *B. amyloliquefaciens* strains GA1 and FZB42 are difficidin 10, macrolactin 12, and bacillaene 11 [59,171,172]. There is an inclusive diversity in the structure and function of these biomaterials due to the modular synthesis. Therefore, it can be subjected to post-translational processing and modifications in the pharmaceutical industry for the designing and exploiting of new drugs or drug precursors [36,59,173]. In addition, *B. subtilis* and its mutants have a potential capacity for the industrial production of riboflavin, guanosine, inosine, and folic acid using the purine biosynthesis pathway [174]. Moreover, *B. subtilis* and *B. pumilus,* with a deficiency in their transketolase or D-ribulose-5-phosphate 3-epimerase, can produce D-ribose. This carbohydrate is commonly used as a taste enhancer in food and feed industries, pharmaceuticals, and muscular painkillers [175]. One of the other biomaterials whose production pathway was reported in some *Bacillus* species, such as *B. licheniformis* and *B. subtilis,* is polyglutamic acid, an anionic homopolyamide, which is edible, biodegradable, and soluble in water. This polymer can be used as a thickener agent, drug carrier, cryoprotectant agent, hydrogel, feed additive, or heavy metal absorbent in many fields [176,177].

It is known that the genus *Bacillus* is a valuable factory for enzyme production. Among those enzymes, extremozymes have an outstanding place in the industries. They are enzymes that are resistant to various and severe environmental factors such as acidic or alkaline conditions, high and low temperatures, or resistance to salinity, oxidizing agents, and detergents. For example, *B. pumilus*, *B. amyloliquefaciens*, *B. licheniformis*, *B. mojavensis,* and *B. subtilis* strains can produce alkaline serine proteases that have fine thermostability and optimal pH 9–12. These properties make them compatible for application in detergents, the leather industry for the dehairing process, silver recovery from X-ray films, etc. Bioengineering and optimization protocols have simplified extremozyme production (e.g., the protease of *S. clausii* and *Bacillus pseudofirmus* (now *Alkalihalophilus pseudofirmus*)) and improved enzyme properties such as pH activity ranges, substrate specificity, binding capacity, and stability. However, it is necessary to consider the disadvantages of these transgenic biomaterials [55,178].

In the following sections, we focused on the other genera of the order *Bacillales* and grouped various families based on the common characteristics, e.g., spore formation or tolerance to extreme conditions for their better understanding as well as applications except for the genus *Bacillus* that was discussed separately.

## 4. Spore-Forming Genera of *Bacillales*

The sporulation in bacteria is a resistance and survival response whenever the environmental conditions are not favorable. In the order *Bacillales*, almost most of the families and genera are able to sporulate. However, some asporogenous genera do not form spores, and there are few genera whose sporulation is not observed under routine laboratory conditions. Therefore, if the spore is not distinguished by microscopy, it will not be a good enough reason to call a strain non-spore former. Nearly sixty conserved genes are essential for sporulation. Any mutations affecting these genes may result in the loss of sporulation ability in a spore former [30]. Spore-forming genera of this order belong to the families *Alicyclobacillaceae*, *Bacillaceae*, *Caryophanaceae*, *Desulfuribacillaceae*, *Paenibacillaceae*, *Pasteuriaceae*, *Sporolactobacillaceae*, and *Thermoactinomycetaceae*. The family *Bacillaceae* includes most of the spore-forming genera in comparison to others [2,14]. Apart from the pathogenic spore-forming species, other members have attracted scientists’ attention to consider spore-forming models, DNA transmitters to space, carriers in surface display systems, biocontrol agents, enzyme factories, etc. [30]. Here, we focused on spore-forming genera (Supplementary Data; Table S1) and explained their significance in different areas, excluding the genus *Bacillus* considered previously. Moreover, some spore-forming genera with special abilities will be explained as extremophiles or polyextremophiles.

### 4.1. Alicyclobacillaceae Genera

Five genera are allocated to this family, *Alicyclobacillus*, *Effusibacillus*, *Kyrpidia*, *Sulfobacillus*, and *Tumebacillus,* which mostly form ovoid endospores in terminal or subterminal positions of swollen rod-shaped cells. Among these genera, *Alicyclobacillus* has special significance; therefore, it is one of the main spoilage causative agents in the beverage industry and its ingredient suppliers. This spore-forming rod-shaped genus is heat and acid-resistant (closely 100 °C due to resistant spores and pH range 2–6), which can tolerate the pasteurization process and is able to grow and produce phenolic odor such as 2-methoxyphenol (guaiacol) and halophenols. However, there are not any pathogenic species within this genus up to now. They can utilize sugars and produce acid. The most important species concerned with unwanted odorous problems of spoiled beverages are *Alicyclobacillus acidoterrestris* and *Alicyclobacillus acidocaldarius*. Formerly, this strictly aerobic genus was classified as *Bacillus; whereas,* later, molecular approaches and its acid- and thermophilicity distinguished it from the genus *Bacillus*. Another characteristic that differentiates *Alicyclobacillus* is the existence of alicyclic radicals in the structural fatty acids that may contribute to condensing membrane lipids (low membrane fluidity) against high temperatures [2,179].

It is important to understand the physiology and resistance properties of *A. acidoterrestris* to prevent economic damage in commercial juice-based beverages. Studies on the AB-1 strain of this species showed that it can survive thermal processing conditions (90–95 °C for 30–60 s), resulting in the viability of the strain in pasteurized beverages. (Spore D-value of this species varies from 0.06 to 5.3 min at 95 °C). Other studies have indicated that spores of this species have greater heat resistance whenever the content of soluble solids (SSC) increases. The pH, growth temperature, and cell density (more thermo-protective proteins) are other effective factors that have a positive correlation with heat resistance. Therefore, it is necessary to find affordable ways to decrease the heat resistance of the spores. One of these ways refers to the usage of the agents which may reduce spore heat resistance, such as lysozyme, organic acids like benzoic acid, emulsifiers (sucrose-based), or nisin. It should be noted that lysozyme is not able to penetrate across the spore layers but owning to the permeability changing, it can meaningfully affect spore heat resistance. Moreover, supercritical CO_2_ is an effective treatment that explodes and deforms spores. An aqueous form of chlorine dioxide is frequently used to sanitize fruits, equipment, and containers in the production line of juice factories to reduce the probability of bacterial cells or spores in the final products. It seems that a combination of two or more inhibitory treatments can be applied to control most spore and vegetative cells in juices and reduce spoilage in this industry [179,180]. Regarding other genera of this family, there are not any interesting sources indicating their biotechnological or industrial applications. However, *Kyrpidia* can be considered a polyextremophilic genus that can grow under high acidity and temperature with the ability of polyhydroxyalkanoate (PHA) production [181]. Moreover, there are some reports regarding the *Sulfobacillus* species that can contribute to chalcopyrite, arsenopyrite, and pyrrhotite bioleaching [182,183,184,185].

### 4.2. Bacillaceae Genera

The most important genus of this family, *Bacillus,* was considered in this review separately. Many genera of the family *Bacillaceae* can be studied as extremophilic or polyextremophilic microorganisms. Most of the genera can form spores with a few exceptions classified in the genus *Bacillus* till the 1990s, but later, based on new taxonomic approaches, new taxa were arranged, whereas 126 validly and not validly published genera composed of one or more species belong to this family up to now [3,14] (Supplementary Data; Table S2). Considerable genera with apparent industrial applications based on their various extraordinary abilities will be discussed in the related sections.

### 4.3. Paenibacillaceae Genera

This family includes 14 spore-forming genera; *Brevibacillus* is one of the most fascinating ones. This genus has been considered a biocontrol agent against mycotoxigenic *Fusaria* and other phytopathogens [186,187]. Mycotoxins are a serious global problem regarding nutritional safety and the economic issues of agriculture. Various fungal species can produce these toxins that commonly toxify various cereals like maize, wheat, corn, and rice. Whenever contaminated grains are consumed by humans or livestock, they may have highly destructive effects [187]. Hence, it would be better to prevent mycotoxins production by easier methods, such as biocontrol agents, rather than using a difficult detoxification process of cereals. Many species of *Brevibacillus* have been considered biological resources for the production of various enzymes, especially cyclodextrin glycosyltransferase used in cosmetics, pharmaceutical, and food industries, serine proteases, biomaterials caused improvement of plants growth or nodulation of roots, antimicrobial peptides, insecticidal agents, extracellular polysaccharide substances (EPSs), polyhydroxyalkanoate copolymers, L-amino acids, etc. [188,189,190,191]. Additionally, *Brevibacillus brevis* strains can degrade many organic compounds such as triphenyltin, pyrene, triphenyl phosphate, or textile dyes, and their high resistance to heavy metals makes them the right choice to use in contaminated sites [192,193,194,195].

The type genus of *Paenibacillaceae* is *Paenibacillus* consists of important species and strains associated with nitrogen fixation, iron acquisition, enzymes, EPSs production, and desulfurization [196,197,198,199]. There are apparently more than 3000 articles about industrial and biotechnological applications of this genus, so it is impossible to discuss all the details in this review. *Aneurinibacillus* belonging to this family has been selected as a model to study S-layer properties during the last few years. It is a thermo- and acidotolerant spore-forming genus whose S-layer consists of glycoprotein subunits oriented in a square matrix. Two main required components for S-layer production in *Aneurinibacillus thermoaerophilus* are dTDP-3-acetamino-3,6-dideoxy-α-D-galactose and GDP-rhamnose, nucleotide activated sugars [200]. Moreover, gramicidin S production has been reported in *Aneurinibacillus migulanus* strains, thus providing a new source of this antibiotic for various purposes, particularly in the treatment of plant diseases [201,202].

### 4.4. Other Spore-Forming Genera and Their Significance

It is shown that there is a massive diversity among spore-forming genera able to survive in very severe conditions and produces a variety of biomolecules applied for particular purposes. In addition to previously discussed spore-forming genera, the families *Caryophanaceae*, *Sporolactobacillaceae*, *Thermoactinomycetaceae*, and *Pasteuriaceae* include genera that may have special biotechnological potentials. *Pasteuria* is the only genus of the family *Pasteuriaceae* considered the biocontrol for invertebrates such as nematodes since it is an obligatory parasitic genus [203,204]. Another possible biocontrol agent that can be referred to as *Fictibacillus* species is a spore-forming genus from the family *Bacillaceae* and includes some species that produce toxic compounds against root-knot nematodes causing infectious disease of crops and make economic losses [205].

*Sporolactobacillus* belonging to the family *Sporolactobacillacae* is known as a homofermentative genus for the production of D-lactic acid. This kind of acid is the precursor of polylactic acid (PLA). The usage of PLA instead of petroleum-derived plastics can decrease synthetic and hazardous materials causing destructive environmental problems. Nowadays, scientists are optimistic about replacing eco-friendly biomaterials with synthetic ones. Therefore, it seems that finding natural and renewable resources for this purpose is certainly significant [206,207]. Another species for a novel biomolecule production is *Mechercharimyces asporophorigenens* YM11-542 from the family *Thermoactinomycetaceae*, which is a mesophilic spore-forming species isolated from the marine environment and can produce a cytotoxic thiopeptide anticancer named urukthapelstatin A. This biomolecule has an IC_50_ value of 12 nM for A549 lung cancer cells [208]. Finding new species and genera from marine sources with very specific abilities reveals a robust metabolic diversity among these microorganisms that may not be found among terrestrial ones. Thermophilic genera of this family, like *Thermoactinomyces,* include species with high enzymatic activities, but *Thermoactinomyces vulgaris* has been considered a causative species of farmer’s lung disorder in addition to *Saccharopolyspora rectivirgula* [209]. Therefore, it is a critical issue to assure about the non-pathogenicity of a strain when applied for industrial and biotechnological goals. Moreover, it should be mentioned that some of the non-spore-forming genera belong to spore-forming families. One example is the genus *Kurthia* assigned to *Caryophanaceae*, a spore-forming family, which can degrade organic compounds and produce extremozymes like proteases [210,211].

## 5. Halophilic Genera of *Bacillales*

Halophiles are referred to as a group of organisms that love salt for their growth and habitation. They usually live in hyper-saline environments, including saline lakes, salterns, saline soils, or saline foods. Most of them belong to the Archaea domain, but there are many bacterial genera and some algae, as well as protozoa, with this property [212]. Generally, bacterial strains are almost moderate or slight halophiles; the members of *Bacillales,* such as *Halobacillus*, *Salibacillus*, and some species of the genus *Bacillus,* are no exception [213]. The main problem of halophiles is intracellular dehydration due to high extracellular salt concentrations. To become accustomed to these conditions, they accumulate inorganic ions such as sodium, potassium, and chlorine. This strategy does not allow losing water by the cell. However, under these conditions, cellular proteins should expose to acidic surfaces to interact with inorganic ions and be stabilized. Another advantageous method for coping with cytoplasm desiccation in halophiles is related to the polar organic osmolytes such as trehalose, proline, glycerol, glutamate, and glycine-betaine [214].

### Potential and Industrial Applications

In traditional biotechnology, we use stainless steel reactors, intricate sterilization processes, expensive up and downstream processing, a high amount of freshwater, and skilled labor that increase costs and capital investments. They caused the bioproducts not to compete with equivalent chemical products based on petroleum. The main reason for the exorbitant costs is related to overcoming the contamination of bioproducts and bio-producers. To confront these issues, biotechnologists try to conduct bioprocesses under open (unsterile) and continuous conditions. Meanwhile, resistant microorganisms are the main part of this procedure [215]. Based on the halophilic property and other special abilities, *Halobacillus* and some species of the genus *Bacillus*, *Lentibacillus, Salimicrobium*, *Planococcus,* or *Virgibacillus* are appropriate candidates for industrial applications. These halophilic genera can produce extremozymes and biodiesels, ferment salty foods, and degrade pollutant compounds in saline environments [216,217,218,219]. Moreover, *Gracilibacillus*, a halotolerant genus, seems applicable as it can produce extremozymes [220,221]. Among halophilic genera, there are many types of research focused on *Halobacillus* because its species are virtuous sources for the production of glycine betaine compatible solute, Cl-1 biosensor, halo-stable serine proteinases, amylases, bioflocculants, antifungal cyclopeptides, etc. [221,222,223,224]. Moreover, it has been reported *Halobacillus trueperi* and two species of *Marinococcus* precipitate carbonates as calcites at different salinities. This process may play a significant role in the biomineralization of carbonates [225,226]. In addition, *Marinococcus,* which is a moderately halophilic and non-spore-forming genus of the family *Bacillaceae,* has been considered for the commercial production of ectoine [227]. *Rummeliibacillus stabekisii*, a halotolerant species from the family *Caryophanaceae*, shows biomineralization activity like *H. trueperi* [228]. Recent research indicates that the halophilic enzymes can be practical for the bioconversion of lignocellulosic biomass into biofuels. In this process, laccases have a key role as they are responsible for the delignification and detoxification of phenolic compounds interfering next steps (cellulose digestibility and extraction) of biofuel production. However, this treatment has very harsh conditions, and using a highly stable laccase is a great help to have a green operation. It would decrease the usage of synthetic surfactants and organic solvents. Furthermore, halozymes are very selective, particularly in the presence of ionic materials. Thus, using halophilic laccases can be a suitable candidate for the bioconversion of lignocellulosic materials. One of these halophilic bacteria is *Aquisalibacillus elongatus* from the family *Bacillaceae,* which produces a very resistant laccase. Furthermore, it can produce a bioactive pigment with biomedical and food industry potentials [221,229,230,231].

## 6. Thermophilic and Psychrophilic Genera of *Bacillales*

Temperature is a significant environmental factor that affects life. Heat and cold-adapted microorganisms are called thermophiles and psychrophiles, respectively. Interestingly, thermophiles were the first extremophiles researchers focused on and studied in detail. Afterward, other extremophiles were discovered and attracted great attention [214,215]. *Psychrobacillus* and *Geobacillus* from *Caryophanaceae* and *Bacillaceae* families are important psychrophilic and thermophilic genera, respectively, which can be applied in various fields [214,232].

### Industrial Applications

Both thermo- and psychrophilic bacteria can produce extracellular enzymes that hydrolyze various compounds and are considered as a frontline in advanced biotechnological industries. Psychrophiles are obviously selected for the degradation of the polymeric contaminants at low temperatures with minimum energy usage. There are applicable cold-active enzymes such as malate dehydrogenase, lipase, iso-merase, xylanase, chitinase, and citrate synthase, while protease is considered an imperative group among them [233]. Recent progress in thermophilic bacteria provides fascinating results, making them substitutes for serving in contaminant-free processes. Furthermore, they are habitually resistant to other extreme conditions, such as low or high pH, organic solvents, and chemical agents. Additionally, they can produce a wide range of thermostable enzymes [215,233,234]. Moreover, thermophiles are related to microbial fuel cells containing anodic biofilm-forming communities with the ability of electrons to transfer to the solid phase to produce electricity. Recently, culture-based and unculturable methods demonstrated the presence of some members of the order *Bacillales,* such as *Geobacillus,* in these communities [235].

*Geobacillus* can be categorized as a polyextremophilic genus because of its resistance to many severe environmental factors. This endospore-forming genus belongs to the family *Bacillaceae* and includes 12 validly published genera with correct names that have impressive abilities [2]. They can utilize various substrates such as hydrocarbons as carbon and energy sources that can be useful for the bioremediation of oil spots in the environment. Furthermore, this genus can produce several enzymes, such as thermostable amylases, xylanases, cellulases, lipases, proteases, and endonucleases, including extraordinary commercial applications. The genus *Geobacillus* has diverse metabolic pathways that result in the production of bacteriocins, ethanol as biofuel, and many other secondary metabolites [236,237]. However, it should not be neglected that some species of this genus, such as *Geobacillus stearothermophilus*, are among the main causes of decomposition in milk or canned foods, although no infectious disease has been reported [2]. A similar thermophilic genus to *Geobacillus* has been described recently and named *Parageobacillus*. It was shown that *Parageobacillus thermoglucosidasius* can be a suitable potent species for the production of biohydrogen via utilizing CO, whereas its final yield is 1.08 H_2_/CO [238].

Thermotolerant and thermophilic genera of the families *Paenbacillaceae*, *Sporolactobacillaceae*, and *Thermoacinomycetaceae* have shown substantial abilities to be used in advanced biotechnology such as *Cohnella* with the capability of nitrogen fixation and the production of thermostable degrading enzymes, e.g., xylanase, chitinase, and agarase. [239,240]. *Thermobacillus* is another example of the family *Paenibacillaceae* that can produce thermostable enzymes with a high hemicellulolytic activity which, in turn, may be used in the production of second-generation biofuels [241]. Various researchers have highlighted xylanases’ role in the production of biofuels from renewable resources. However, another notable enzyme, xylulokinase, which is essential for the metabolizing of the liberated D-xylose, has not been studied in thermophilic microorganisms in detail. This enzyme has been found in a thermophilic species of *Bacillaceae* named *Saccharococcus caldoxylosilyticus* (now *Parageobacillus caldoxylosilyticus*) strain S1812, and despite previously described xylulokinases, it is not inducible by xylose. Therefore, the routine process of biofuel production can be changed to achieve a higher yield [242]. In addition to the mentioned genera, *Thermoflavimicrobium,* a thermophilic genus of the family *Thermoactinomycetaceae,* attracted researchers’ interest due to its capability to produce a novel and useful iso-merase for the commercial production of D-mannose [243]. Moreover, *Novibacillus thermophilus*, belonging to this family, is a proper candidate for the bioremediation of hot wastewaters containing azo dyes [244,245].

It seems that most psychrophilic genera within the order *Bacillales* belong to the family *Caryophanaceae*. Although psychrophiles and psychrotolerants distribute among all families of this order, they do not form a distinctive group. It is noteworthy to say that little information about biotechnological and industrial applications of *Bacillales* psychrotolerants and psychrophiles has been mentioned up to now. The investigation and exploration of these fascinating bacteria continue to clarify more details on how to apply them for various purposes. Recently, a psychrotolerant and spore-forming genus, *Sporosarcina*, has been used for microbially induced carbonate precipitation (MICP) [246,247,248]. In addition to the psychrophilic genera of the family *Caryophanaceae*, *Ureibacillus* is a thermophilic genus assigned to this family. This genus has seemingly presumable applications for biofuels, enzymes, and nanoparticle production [249,250,251].

## 7. Extremophiles and Polyextremophilic Genera of *Bacillales*

Extremophiles are organisms able to survive under hostile environmental conditions such as different acidity, temperature, salts, and heavy metals. Polyextremophilic property refers to extremophiles that can withstand more than one intense state, such as thermoacidophiles living in acidic hot springs or halophiles living in low-oxygen areas. Studying these microorganisms provides a lot of information to understand how to use them for various applications. One of these attractive fields relates to astrobiology, exploring the possibility of life in the solar system [214]. In the order *Bacillales*, some genera can be called extremophiles and polyextremophiles, but it is not possible to create an exact boundary to define them as extremophile or polyextremophile. Due to the importance of halophilic, thermophilic, and psychrophilic genera, we considered them in the previous sections. Here, we discussed other extremophiles and polyextremophiles of the order *Bacillales*. A list of (poly)extremophilic genera of this order has been shown in the Supplementary Data, Table S3.

### 7.1. Acidophilic Genera and Their Applications

Acidophiles can grow under high acidity (pH less than 3). Even though many bacterial strains grow in the range of neutral pH, they can grow at the lower pH range (acidotolerants). Acidophiles use several cellular strategies to confine the influx of H^+^. One of these strategies is to decrease cell membrane permeability to adjust intracellular pH. In addition, they have a metabolic preference for the oxidation of compounds producing H_2_ instead of H^+^ and can respond to slight acidification by cytoplasm buffering. Therefore, acidophiles and their products have extraordinary properties making them remarkably used in advanced industries applying low pH conditions in their processes. Moreover, these conditions prepare an unfavorable environment for the contaminant microorganisms [214,215]. It should be considered that all acidophiles are not necessarily useful for the industry e. g., *Alicyclobacillus* is a spoilage agent of fruit juices [179]. In the order *Bacillales*, most genera grow under neutrophilic conditions, but moderately acidophiles are widespread in different families. *Alicyclobacillus* is the most known acidophilic genus in this order. *Kyrpidia* and *Hydrogenibacillus* are moderately acidophilic genera that belong to *Alicyclobacillaceae* and *Bacillaceae*, respectively; however, it is possible to find only one strain that can grow under low pH conditions [2,252].

Interestingly, most extremophiles can tolerate other harsh conditions, such as high temperatures. *Alicycobacillus* or *Kyrpidia* are thermophilic genera with the capability to tolerate low pH. *Kyrpidia* is a thermoacidophilic genus that grows on CO and CO_2_ as sole carbon sources using H_2_ as the electron donor [253,254]. Recently, it has been reported *Kyrpidia spormannii* FAVT5 showed a high affinity to H_2_, which is an exception in the phylum Firmicutes [214]. Moreover, this species has been used as a novel biocatalyst in the electrosynthesis processes [255].

### 7.2. Alkaliphilic Genera

In contrast to acidophiles, microorganisms living in alkaline conditions (more than pH 9) are called alkaliphiles. Furthermore, some microorganisms can tolerate such low acidity and group as alkalitolerants. Alkaliphiles can be found in alkalithermal waters, soda lakes, or shallow hydrothermal vents. They need to increase the H^+^ influx to drive the power plant of the cell (ATP synthesis) and neutralize the cytoplasmic area. For this purpose, they use sodium or potassium-proton anti-porters to transport monovalent cations out and proton in, respectively. Furthermore, the acidic composition of the cell wall and buffering state of the cytoplasm can help keep them alive under alkaline conditions. Because of the astonishing properties of alkaline lovers, they are very promising candidates for the biorefining of polymeric carbohydrates (cellulose or hemicellulose) [42,214,215].

*Amphibacillus* is a facultative anaerobic spore-forming genus belonging to the family *Bacillaceae* and can grow under alkaline conditions (pH 8–10) [256]. This genus consists of eight validly published species with correct names. Some strains of *Amphibacillus* have the opportunity to consider as applicable strains, such as *Amphibacillus* sp. strain C40, isolated from an old indigo fermentation liquor that reduces this dye during its fermentation process [257]. Moreover, this dye is reduced by *Fermentibacillus polygoni*, a moderately alkalophilic spore-forming genus of the family *Bacillaceae* [258]. Another strain of *Amphibacillus* sp. can synthesize PHA polymers from ammonium-rich wastes, which are an important issue nowadays for resource recovery [259]. Strain KSUCr3 of this genus is confirmed as a heavy metal-reducing bacterium under extreme alkaline conditions. It is very tolerant to the high concentration of chromium, nickel, molybdenum, cobalt, manganese, zinc, copper, and lead and reduces them rapidly. Hence, it could be an efficient detoxifier of heavy metals existing in dangerous wastes. For example, this strain reduces 237 μMh^−1^ of Cr (VI) continuously under optimum conditions. It is one of the most rapid rates among other microorganisms [260]. Furthermore, this genus is a reliable source for the extremozymes such as glucoamylopullulanse produced by strain NM-Ra2. This kind of amylase has distinct amylolytic activity under high pH and temperature. Moreover, it has considerable stability in organic solvents, metallic ions, and high salt concentrations. Moreover, it can act on a wide range of substrates. These critical properties are very important that make this strain suitable to be used in the starch industry [261].

### 7.3. Polyextremophilic Genera

Being able to face several harsh conditions simultaneously does not seem to be simple, but polyextremophiles manage their life by using survival strategies and synergistic adaptations to cope with the problems. This ability makes them more applicable in various industries. For example, thermoacidophiles can be used for lignocellulosic hydrolysis and biofuel production. Moreover, studying these microorganisms can reveal the fundamental points used for extraterrestrial life simulation by astrobiologists. Based on factors that polyextremophiles tolerate, they can be sorted into many groups: 1, a combination of pH and temperature; 2, salinity and temperature; 3, pressure and temperature; 4, radiation and temperature; 5, pH and salinity; 6, pH and pressure; 7, pH and radiation; 8, salinity and pressure; 9, desiccation and radiation; 10, temperature, desiccation, and pressure; and 11, pH, salinity and desiccation [214,262]. This classification helps us discuss polyextremophilic genera of the order *Bacillales* without any confusion. However, it should be considered that some groups may not have proper examples within this order.

Depending on industrial demands, different types of polyextremophiles and their products can be selected. Therefore, using wide-spectrum strains improves their functionality in each field they have been used. One of the first interesting genera discussed here is *Exiguobacterium*, a versatile member of the order *Bacillales* whose parent taxon is *Bacillaceae*. This pigmented, non-spore-forming bacterium can grow under very diverse environmental conditions. It is possible to isolate its strains from various places, including soils, glaciers, seawaters, sediments, hydrothermal vents, and forests. *Exiguobacterium* strains have been applied to produce resistant hydrolyzing enzymes such as xylanase, cellulase, tannase, or mannanase, which are involved in the biofuel production processes. Other extremozymes like lipases, amylases, and proteases have been reported among these strains. Furthermore, this genus is cable of remediating and degrading heavy metals, dyes, aromatic as well as volatile hydrocarbons. Likewise, some strains of this genus can promote plant growth via the production of indole acetic acid, siderophores, and hydrogen cyanide [263,264].

The next genus is a knallgas (hydrogen oxidizing) bacterium classified as *Bacillus* due to low chemotaxonomic and whole-genome information; however, it is currently identified as *Kyrpidia*, a spore-forming thermoacidophile isolated from sulfataric areas and includes only two species, *Kyrpidia tusciae,* and *Kyrpidia spormannii* [254]. These species can grow autotrophically and heterotrophically. *K. tusciae* can utilize 2-hydroxyisobutyric acid as the sole source of carbon and energy at 55 °C, associated with B12-dependent mutase activity. This kind of mutase, due to its high specificity, has the potential to be used for the synthesis of stereospecific carboxylic acids such as poly (methyl methacrylate) from renewable sources not easily achievable by routine chemical reactions [265].

Among the thermophilic genera, *Aeribacillus* has merely two species, *Aeribacillus pallidus* and *Aeribacillus composti*. *A. pallidus* has remarkably attracted the interest of researchers because of its polyextremophilic characteristics. In general, this species is an alkali- and halotolerant spore former thermophile that can produce exopolysaccharides (EPSs) [266,267]. Although the production of thermophilic EPSs is not a cost-effective process, the produced EPSs have many advantages, including metal adsorption, short fermentation time due to high growth rates, highly thermo-resistant, and stability in the water/oil emulsions. These properties make it a suitable substitute for chemical stabilizers and emulsifiers in the cosmetic and food industries. For example, *A. pallidus* YM-1 and 418 can produce stable EPSs with promising usages as gelling agents, thickeners, suspenders, or coagulants due to their composition, molecular weight, and structural properties [268,269,270]. Another considerable application of *Aeribacillus* is related to thermostable antibacterial peptide production as a preservative agent in foods undergoing thermal processing. Moreover, this potential ability has been reported among other thermophilic genera like *Anoxybacillus* and *Geobacillus* [271]. The halotolerant strain TD1 of the genus *Aeribacillus* is a probable candidate for the industrial production of thermostable pectate transeliminase (pectate lyase) applied as an extractive agent of wine or fruit juices, softener of vegetables, scrubbing agent of cotton, animal feed, pretreating agent of pectinaceous wastes, etc. [272].

Strain TSHB1 of *A. pallidus* is another suitable example of the carbonic anhydrase production used as a catalyst for CO_2_ sequestration. This applicability should be considered to be a cost-effective method for this process [273]. Lastly, researchers have indicated that the genus *Aeribacillus* is reclassified from *Bacillus* and *Geobacillus* and forms a new taxon. It is a potentially suitable microorganism that can degrade *n*-alkanes and polycyclic aromatic hydrocarbons [274,275]. Moreover, it can produce thermozymes such as serine alkaline protease [276,277], xylanase [278], 4-α-glucanotransferase [279], and Cr (IV) reductase [280]. *Bacillus haloalkaliphilus*, another interesting polyextremophile, was reclassified as a newly proposed genus in 2005. *Alkalibacillus* is known as a spore-forming halo- and alkaliphilic genus with a wide range of biotechnological applications, particularly ectoine and extremozyme production [281,282]. These extremozymes include alkali-thermostable proteases and organo-solvent resistant lipases with potential usages in laundry and leather processing, while ectoine can be used in the cosmetic industry and molecular biology as a stabilizer [283]. Moreover, the genus *Anoxybacillus* is an alkalophilic thermophile that has been considered a relatively new source of extremozymes. These enzymes can be used in various fields such as resource recovery of lignocellulosic materials, starch industry, bioremediation of organic compounds, biosorption of heavy metals, biohydrogen production as renewable energy, and production of bioactive compounds [284]. Comprehensively, biohydrogen-producing members phylogenetically are closer to the genus *Clostridium* and use the formate hydrogen lyase (FHL) pathway for this purpose. In comparison to *Clostridium*, H2-producing members of the order *Bacillales* are less susceptible to oxygen and more resistant to heat-shock treatment. Furthermore, they have a versatile metabolic capacity making them utilize a wide variety of substrates and are resistant to extreme pH values and salts [285].

Within the family *Bacillaceae*, *Anaerobacillus*, an anaerobic spore-forming genus with tolerance to high pH values and high salt concentrations, attracted the scientists’ interest due to its ability for bioremediation of environments contaminated by oxyanions of selenium and arsenic. *Anaerobacillus arseniciselenatis,* isolated from the alkaliphilic lake, can reduce arsenate (As (V)) to arsenite (As (III)) while oxidizing lactate to carbon dioxide and acetate. Moreover, it can reduce selenate (Se (VI)) to selenite. Therefore, using the co-culture of *A. arseniciselenatis* and *Bacillus selenitireducens* (now *Salisediminibacterium selenitireducens*) which reduce selenite (Se (IV)) to selenium (Se (0)), can help to convert selenate to elemental selenium [286].

Furthermore, the genus *Lysinibacillus* is a significant and resistant one used for the bioremediation of heavy metals such as arsenic, selenium, and cadmium. Haloalkalitolerant *Lysinibacillus* can utilize hydrocarbons, and also its species can produce antifungal biomaterials, biosurfactants, and Bt-like endotoxins [287,288,289]. Although the family *Staphylococcaceae* has been known for MRSA, *Nosocomiicoccus,* and some virulent species of *Macrococcus*, it includes non-pathogenic polyextremophilic genera such as *Salinicoccus, Abyssicoccus,* and some halotolerant genera like *Jeotgalicoccus* that are interesting for biotechnological applications [229,290,291]. Fascinatingly, the genus *Macrococcus* includes some species with potential industrial applications, like *Macrococcus bovicus,* which is able to produce silver nanoparticles, or *M. caseolyticus,* which is related to the development of flavor in fermented foods [292,293]. In addition to the genera discussed here, there are many other polyextremophilic genera in the order *Bacillales* with exceptional properties that may make them applicable in the future (Table 3).

## 8. Engineered Strains of *Bacillales*

One of the most striking microorganisms from the order *Bacillales* is *B. subtilis* known as GRAS [313]. The production of many different metabolites (organic acids, chemicals, biosurfactants, antibiotics, peptides etc.) has been greatly improved by developing various bioengineered applications (heterologous gene expression, inducible promoter introduction, gene deletion, and transcriptional regulation) in *Bacillus* strains [313,314,315,316,317].

At the core of bioengineering studies, researchers have focused on increasing the activity of microbial strains for efficient and simultaneous use of different types of sugars (glucose, xylose, and arabinose) from lignocellulosic materials [318]. The *xylA* and *xylB* genes encode xylose iso-merase and xylose kinase, respectively, and these genes are regulated by *xylR* (xylose-responsive repressor protein) and carbon catabolite repression in *B.subtilis* [319,320]. However, it was determined that xylose could be transported to the cell via *araE* gene (xylose/arabinose transporter gene) when arabinose is present in the medium, but wild-type *B. subtilis* strain could not consume xylose in media containing only xylose [321]. Thus, the *araE* expression cassette was constructed and then integrated into *B. subtilis*, allowing xylose to be used even in only xylose-containing media [322]. With the effective use of xylose, some chemicals such as acetoin, poly-*γ*-glutamic acid (*γ*-PGA), and biofuels have been effectively produced from different sugar-containing substrates such as glucose, xylose, and arabinose [323,324,325,326,327].

*Bacillus* has a robust expression system frequently used in the production of polymers such as *γ*-PGA and polyhydroxyalkanoate since their strains do not contain lipopolysaccharides [328]. *γ*-PGA is a water-soluble polymer consisting of D- and L-glutamic acid monomers, known as non-toxic, edible, and biodegradable [329]. This polymer is generally synthesized in the presence of glycerol, citrate, and glutamic acid. On the other hand, Halmschlag et al. [330] developed two bacterial strains that can produce *γ*-PGA by consuming glucose. For this, the natural promoter of the PGA synthetase operon was replaced with the strong constitutive promoter P_veg_ or the xylose-inducible promoter P_xyl_. 129% more γ-PGA production was achieved in glucose medium by bioengineered *B. subtilis* strain [330]. In another study of γ-PGA production, Cai et al. [331] focused on improving ATP supply in engineered strains of *B. licheniformis*. The deletion of *cydB* (cytochrome bd ubiquinol oxidase (subunit II)) and *cydC* (ATP-binding/permease protein) and expression of *Vitreoscilla* hemoglobin (VHb) improved ATP-synthetic and nitrate metabolism pathways, enhancing the ATP supply and *γ*-PGA yield [331]. Expression of bacterial hemoglobin (VHb) in heterologous hosts improves oxygen supply to cells and thus improves growth, production of value-added metabolites, and ability to degrade organic compounds [198,332]. Similarly, VHb expression could improve surfactin biosynthesis in *B. subtilis* [333]. Surfactin production was also reported to be increased 6.4-fold by overexpressing the signaling factors encoded by *comX* and *phrC* [334]. The production of hydroxybutyrate, another important biopolymer, can also be increased by metabolic engineering. Akdoğan and Çelik [335] developed a bioprocess using a recombinant *P. megaterium* strain overexpressing the native *phaC* gene (PHA synthase) for excess Poly(3-hydroxybutyrate-co-3-hydroxyvalerate) (PHBV) production from glucose without the addition of any precursors. Cal et al. [328] also inserted three genes (4-hydroxybutyryl-CoA transferase (*orfZ*), succinate semialdehyde dehydrogenase (*sucD*), and 4-hydroxybutyrate dehydrogenase (*4hbD*)) for succinate utilization from *Clostridium kluyveri* into *P. megaterium* under the control of *xylA* promoter, and thus succeeded in more effectively producing (>10% mol) PHBV copolymer from succinate.

Additionally, the production of 2,3-butanediol is a common metabolite in *Bacillus* strains. Researchers have focused on more efficient production of this compound by synthesizing its production in the early logarithmic phase rather than the late logarithmic and stationary phase, depending on the expression of *bdhA* gene (acetoin reductase) [336,337]. Qiu et al. [338] developed an engineered *B. licheniformis* strain to produce the high titters of meso-2,3-butanediol. The strain can normally produce a mixture of D-2,3-butanediol and meso-2,3-butanediol isomers. To eliminate the production of D-2,3-butanediol, two genes, which are glycerol dehydrogenase (*gdh*) and acetoin dehydrogenase (*acoR*), were deleted from wild-type strain to provide adequate flux from acetoin toward meso-2,3-butanediol. Thus, meso-2,3-butanediol was improved by 50% through a double-deletion mutant [338]. Fu et al. [339] also deleted some genes in the metabolite pathways for pure meso-2,3-butanediol production and then cloned the *budC* gene from *Klebsiella pneumoniae*. Thus, an industrial butanediol producer, *B. subtilis*, was developed by replacing *bdhA* in *B. subtilis* with *budC* from *K. pneumonia* [339]. Moreover, Drejer et al. [340] developed a bioengineered strain of *Bacillus methanolicus* to produce acetoin from methanol. For the industrial strain, three different heterologous *alsSD*/*budAB* operons encoding acetolactate synthase and acetolactate decarboxylase were expressed under the control of an inducible promoter. Moreover, overexpression of malic enzyme (*mae* from *G. stearothermophilus*) with isocitrate lyase gene (from *B. methanolicus*) increased acetoin titers by 1.6-fold [340]. Bioalcohol (ethanol, isobutanol, and methylbutanols) production capabilities of *Bacillus* species can be improved by the inactivation of the RelA regulatory protein by additional lysine and cysteine supplementation [341].

Hyaluronic acid (hyaluronan or HA) is a sulfate-free glycosaminoglycan generally produced by highly pathogenic *Streptococcus* species and is mostly used in various industries such as cosmetics, food, and pharmaceuticals [342]. Relevant genes from *Streptococcus* were transferred to GRAS microorganisms such as *Bacillus* for bio-safe production of hyaluronic acid [343,344,345,346,347]. Chien and Lee [346] developed a mutant *B. subtilis* strain by integrating hyaluronic acid production-related genes (*hasA* and *hasB*) from *Streptococcus* into *B. subtilis* or integrating *B. subtilis* itself (*tauD*) into the *amyE* locus. While HA production can only be achieved by expression of the *hasA* gene, the production was better through the co-expression of *hasA* with *hasB* and *tauD*. In addition, the expression of *Vitreoscilla* hemoglobin could also increase both cell density and metabolite production [346]. On the other hand, Jin et al. [348] increased the hyaluronan production from 1.01 g/L to 3.16 g/L through overexpression of some committed genes (*tuaD*, *gtaB*, *glmU*, *glmM*, and *glmS*) and down-regulating the glycolytic pathway. Similarly, mannan production was improved through overexpression of identified committed genes (*manC*, *manB*, *manA* and *pgi*) in *B. subtilis* [349]. On the other hand, different kinds of polysaccharides such as chondroitin and heparosan could be produced by bioengineered *B. subtilis* via integrating related synthases genes derived from *E. coli* and overexpressing *tuaD* in *B. subtilis* [350].

*B. thuringiensis* is the most widely used microorganism for commercial biopesticide production [351,352]. Bioengineering studies are also carried out in order to promote sporulation and toxin production and/or to produce a broader spectrum insecticide of *B. thuringiensis* [351,353,354]. For example, an engineered strain with a broader spectrum of activity against two different insects was developed by incorporating *cryIC* gene (its products are active against *Spodoptera littoralis*) in *B. thuringiensis* (active against *Ostrinia nubilalis*) [353]. Similarly, Yan et al. [354] developed a recombinant *B. thuringiensis* strain by introducing the recombinant plasmid pSTK-3A containing *cry3Aa7* into the wild-type *B. thuringiensis* strain containing the *cry8Ca2* gene. On the other hand, Lertcanawanichakul et al. [355] cloned chitinase genes from *Aeromonas hydrophila* and *N. circulans* into *B. thuringiensis* and obtained 15 times higher chitinase activity. For the products of *B. thuringiensis* to become more toxic, the integration of various genes into bacteria or overexpression of certain genes can be carried out [356,357,358]. Yu et al. [357] integrated two genes, *cyt1Aa* and *cry11Aa*, into *B. thuringiensis* in order to obtain a more toxic product. Doruk et al. [358], on the other hand, increased insecticide production by overexpressing the polyphosphate kinase gene.

## 9. Conclusions and Perspectives

The order *Bacillales* includes 10 validly published families with 218 validly and non-validly published assigned genera that show high physiologic, metabolic, and genetic diversity. Their ecological habitats vary from glaciers to hydrothermal vents, and some of them cannot live without a host. Although recognized pathogenic genera, *Staphylococcus* and *Listeria*, belong to this order, most of the genera within *Bacillales* have been considered as biotechnological resources that can be implicated in several fields, including food and pharmaceutical industries, agriculture, bioremediation of contaminated sites, molecular biology, and medicine. One of the main genera with high capacity and efficacy is *Bacillus*. Species of this two-edged genus can be grouped into pathogenic (*B. cereus* group) and soil (*B. subtilis* group) groups. The latter group encompasses more important industrial strains which display outstanding abilities.

For better understanding, in this review, *Bacillales* genera on the basis of their extremophilicity have been grouped into spore-forming, halophilic, thermophilic, psychrophilic, acidophilic, alkaliphilic and polyextremophilic genera. Like the *Bacillus* genus, every group exhibits more than one property that can be biotechnologically significant and preserve their position as dominant genera in industries. In addition to *Bacillus*, some genera like *Pullulanibacillus* have been confirmed to be used for the production of enzymes, antimicrobial agents, biopolymers, fermented foods, etc. However, as indicated in this review, so many species of the order *Bacillales* are potent resources for providing green industries in the future. One of the reasons that limited the use of these potent strains is related to a lack of knowledge about their physiology and metabolic activities. In addition, some of them are assigned to recently identified genera or families, and therefore, it takes time to make them ready for industrial applications, and future efforts should be on the screening of biotechnological abilities of less-known genera with high resistance to harsh conditions. Moreover, another focus should be dedicated to complete genome technology and metabolomics to discover the adaptation mechanisms used by *Bacillales* genera.

## Figures and Tables

**Table 3:** Industrial and biotechnological potential of some (poly)extremophilic genera in the order *Bacillales*.

Genus Name	Strain Name	Type Strain	Important Properties	Potential Applications	(Poly)Extremophilic Features		Reference
*Aliibacillus*	*A. thermotolerans* strain BM62	Yes	Ammonia oxidation	Reduction of the air pollution caused by ammonia volatilization; Increase nitrate content in the compost	Moderately thermophile		[294,295]
*Alkalilactibacillus*	*A. ikkensis* strain GCM68	Yes	Production of a cold-active β-galactosidase	Heat-labile enzymatic digestions in genetic engineering; An affordable choice for laundry detergents; Food processing at low temperature (lactose hydrolyzing of milk)	Alkaliphile Halotolerant Psychrophile		[296,297]
*Allobacillus*	*A. halotolerans* strain MSP69	No	Production of an extracellular alkaline nuclease	Biocatalyst and flavor enhancer of fish sauce	Halotolerant		[298]
*Caldalkalibacillus*	*C. thermarum* strain TA2.A1	No	Production of a thermostable laccase causing dimerization of a dimeric lignin model compound GGGE	Biocatalyst for delignification and detoxification of lignocellulosic biomass	Thermoalkaliphile		[299,300]
*Filobacillus*	*Filobacillus* sp. RF2–5	No	Production of a halo- and thermostable serine proteinase	Useful for the degradation of fish protein during fermentation at high salt concentrations and might be useful for reduction of the fermentation period	Moderately halophile		[301]
*Oceanobacillus*	*Oceanobacillus oncorhynchi* subsp. *incaldaniensis* strain 20AG	Yes	Production of a novel thermo-alkali stable catalase–peroxidase	Useful for phenol resin synthesis; Bioremediation of anilines and phenols; Lignin degradation in paper industry	Halophile Alkalitolerant		[302,303]
	*Oceanobacillus* sp. PUMB02	No	Production of a halotolerant thermostable lipase	Potential utility in inhibiting biofilm formation in a food processing environment			[304]
*Paralkalibacillus*	*Paralkalibacillus indicireducens* strain Bps-1	Yes	Indigo reduction	Potential use in indigo dying industry	Obligately alkaliphile Halotolerant		[305]
*Virgibacillus*	*Virgibacillus* sp. SK37	No	NaCl-activated Proteinase	Potential starter to improve fish sauce quality	Moderately halophile		[306,307,308]
*Laceyella*	*Laceyella sacchari* strain LP175	No	Thermostable amylase	Raw starch industry; Bioethanol production	Thermophile		[309]
	*Laceyella putida* strain JAM FM3001	No	Highly thermostable and surfactant-activated chitinase	Potential applications in waste management; Biocontrol agent; Pharmaceutical industry			[310]
*Halolactibacillus*	*Halolactibacillus alkaliphilus* MSRD1	No	Antibacterial activity of red pigment	Pharmaceutical industry	Halotolerant		[311]
	*Halolactibacillus miurensis* strain SEEN MKU3	No	Production of exopolysaccharides with antioxidant activities	Potential applications in functional foods; Cosmetic industry; Pharmaceutical industry			[312]

## Data Availability

Not applicable.

## Supplementary Materials

The following supporting information can be downloaded at: https://www.mdpi.com/article/10.3390/microorganisms10122355/s1, Table S1: Spore-forming genera of the order *Bacillales*; Table S2: The allocated genera to the family *Bacillaceae*; Table S3: list of (poly)extremophilic genera in the order *Bacillales*. References [20,22,26,256,258,295,297,359,360,361,362,363,364,365,366,367,368,369,370,371,372,373,374,375,376,377,378,379,380,381,382,383,384,385,386,387,388,389,390,391,392,393,394,395,396,397,398,399,400,401,402,403,404,405,406,407,408,409,410,411,412,413,414,415,416,417,418,419,420,421,422,423,424,425,426,427,428,429,430,431,432,433,434,435,436,437,438,439,440,441,442,443,444,445,446,447,448,449,450,451,452,453,454,455,456,457,458,459,460,461,462,463,464,465,466,467,468,469,470,471,472,473,474,475,476,477,478,479,480,481,482,483,484,485,486,487,488,489,490,491,492,493,494,495,496,497,498,499,500,501,502,503,504,505,506,507,508,509,510,511,512,513,514,515,516,517,518,519,520,521,522,523,524,525,526,527,528,529,530] are cited in the supplementary materials.
